# Study protocol for a theory-informed randomized controlled trial of a lifestyle and sleep intervention to improve quality of life and physical activity in inactive adults: the *SleeP exercIse nutRition heALth+* SPIRAL+ study

**DOI:** 10.1186/s12889-026-26959-4

**Published:** 2026-03-13

**Authors:** Monique Mendelson, Marie Coudurier, Stéphane Doutreleau, Michel Guinot, Patrice Flore, Sébastien Bailly, Sébastien Baillieul, Marie Destors, Renaud Tamisier, Jean-Louis Pépin, Damien Tessier, Matthieu Roustit, Marc Manceau, Aïna Chalabaev, Samuel Vergès

**Affiliations:** 1 Univ. Grenoble Alps, HP2 Laboratory, Inserm U1300, Grenoble, France; 2 Sports & Pathology Department, CHU Grenoble Alpes, Echirolles, France; 3 Univ. Grenoble-Alpes, SENS, Grenoble, France; 4 Laboratoire Du Sommeil, Pole Thorax et Vaisseaux, CHU Grenoble Alpes, Grenoble, France; 5 Inserm CIC 1406, Grenoble Alps University Hospital, Univ. Grenoble Alps, Grenoble, France

**Keywords:** sleep, physical activity, lifestyle intervention, quality of life

## Abstract

**Background:**

Sleep, physical activity, and diet are key determinants of health, each independently associated with chronic disease risk and mortality. These behaviors also interact: insufficient sleep can impair physical activity and dietary choices, while regular exercise and healthy diet promote better sleep. Although lifestyle interventions commonly target physical activity and diet, few simultaneously address sleep. Emerging evidence suggests that even small improvements across all three behaviors can reduce all-cause mortality, highlighting the potential of multi-behavior approaches. However, the effects of a lifestyle intervention simultaneously targeting sleep, physical activity, and diet on quality of life and physical activity levels in inactive adults remain largely unexplored.

**Methods:**

The SPIRAL+ study is a single-center, randomized controlled trial conducted in France, among non-exercising adults aged 18–80 years. Participants (*n* = 201) will be randomized to one of three groups: (1) lifestyle intervention (physical activity and diet) (2), lifestyle plus sleep intervention, or (3) control. Assessments will be conducted at baseline, 6 months (post-intervention), and 12 months. The primary outcomes are health-related quality of life (EQ-5D-5 L) and daily step count (measured by accelerometer) assessed immediately after the 6-month intervention. Secondary outcomes include whether intervention effects are sustained at 12 months, along with markers of physical fitness (cardiorespiratory fitness, body composition, handgrip strength), physical activity and sedentary behavior (accelerometry), and sleep (home-based sleep test, actigraphy, and validated questionnaires). Additional self-reported outcomes will cover diet, mental wellbeing, motivation, quality of life, and psychological constructs related to health behavior change. A qualitative component will explore barriers and facilitators to adherence through semi-structured interviews.

**Discussion:**

This trial will evaluate whether adding a sleep component to a lifestyle intervention improves quality of life and physical activity levels in inactive adults. If effective, the findings will support the integration of sleep into multi-behavior interventions to enhance health outcomes and inform future public health strategies.

**Trial registration:**

Clinical Trials NCT06424847.

**Supplementary Information:**

The online version contains supplementary material available at 10.1186/s12889-026-26959-4.

## Introduction

Sufficient sleep, regular physical activity, and a balanced diet are fundamental pillars of health and well-being, each contributing to the prevention of chronic diseases and premature mortality [1, 2].

Inadequate sleep has been linked to metabolic and cognitive dysfunction, driven by mechanisms such as insulin resistance, inflammation, and dysregulation of appetite-related hormones [3, 4]. Physical inactivity is a well-established risk factor for numerous chronic conditions [1], while meeting physical activity recommendations is associated with a significantly lower risk of all-cause mortality [5, 6]. Similarly, poor dietary habits, including excessive calorie intake and nutrient-poor food choices, contribute to the development of common non-communicable diseases, accelerate biological aging, and increase the likelihood of early mortality [7, 8].

Beyond their individual impact, these three behaviors are intricately linked. Sleep deprivation can lead to reduced physical activity due to fatigue, while engaging in regular exercise has been shown to improve sleep quality [9–11]. Likewise, insufficient sleep disrupts appetite regulation, influencing food intake and dietary choices [12] and, conversely, an unhealthy diet can impair neurotransmitter activity and sleep patterns [13].

Emerging evidence shows that even modest improvements across these domains yield measurable benefits. In a recent prospective cohort, small increases in sleep duration, physical activity, and diet quality were associated with a 10% lower risk of all-cause mortality [14]. These findings highlight the importance of developing effective interventions that simultaneously target these sleep, physical activity and diet to optimize health outcomes.

Previous studies have primarily evaluated lifestyle interventions targeting physical activity and diet in primary prevention. A recent meta-analysis showed that, compared to usual care, such interventions reduced blood pressure, total cholesterol, BMI and waist circumference [15]. Evidence also indicates improvements in health-related quality of life among adults with metabolic syndrome [16]. Interventions focusing on sleep have also been tested; for example, a recent randomized controlled trial combining physical activity and sleep strategies improved sleep quality in middle-aged adults with poor baseline sleep [17]. However, to our knowledge, no study has yet examined the combined effects of an intervention targeting physical activity, diet, and sleep on quality of life and physical activity levels in inactive adults.

Taken together, these findings underscore the need for innovative interventions that move beyond single-behavior approaches and address the interconnected nature of sleep, physical activity, and diet. Designing such multi-component programs requires not only targeting these behaviors simultaneously but also ensuring that participants are supported in adopting and maintaining changes over time. To this end, drawing upon robust theoretical frameworks [18, 19] is essential to guide intervention content, enhance adherence, and maximize the likelihood of sustainable health improvements.

### Theoretical framework

The lifestyle intervention targeting physical activity and diet is grounded in self-determination theory (SDT) and affectivism. SDT highlights the role of autonomous motivation, when behavior is driven by choice, values, or enjoyment, in sustaining long-term engagement [20–23]. By supporting the basic needs for autonomy, competence, and relatedness, the intervention seeks to foster autonomous motivation. In addition, research on affective responses to physical activity indicates that positive experiences, especially at peak and final moments (“peak-end rule”), can shape remembered and anticipated pleasure, reinforcing future participation [24].

The sleep component is informed by the theory of planned behavior (TPB) and the health action process approach (HAPA). TPB emphasizes the role of attitudes, norms, and perceived behavioral control in shaping intentions [25, 26], while HAPA addresses the well-documented intention–behavior gap by integrating self-regulatory processes such as action and coping planning [27]. These frameworks guide the selection of evidence-based behavior change techniques to promote sustainable improvements in sleep health.

### Study objectives

The SPIRAL+ study is designed to address this evidence gap by evaluating the effectiveness of a lifestyle intervention that integrates sleep, physical activity, and diet.

The primary objective of the study is to determine whether adding a structured sleep component to a standard lifestyle program improves health-related quality of life and physical activity (step count) in physically inactive adults, compared to a lifestyle intervention without sleep support. These primary outcomes will be assessed immediately after completion of the 6-month intervention.

The secondary objectives are to examine whether the effects on quality of life and physical activity are sustained at 12 months, to evaluate additional health outcomes such as physical fitness (cardiorespiratory fitness, body composition, handgrip strength), sedentary behavior, sleep quality and duration, dietary habits, mental wellbeing, vitality, and motivational or psychological constructs, and to explore barriers and facilitators to adherence through a qualitative component that includes semi-structured interviews with participants.

## Methods

### Study design

The SleeP exercIse nutRition heALth+ (SPIRAL+) study is a single-centre, three-arm randomized controlled trial (RCT) designed to evaluate the effects of a lifestyle intervention (physical activity and diet), a lifestyle and sleep intervention, and a control condition in non-exercising adults aged 18–80 years. Participants will be recruited through passive methods (advertisements in the community and/or on social media) or active methods (through word of mouth or referral by a health professional). Eligible participants will be randomly allocated (1:1:1) using a computer-generated sequence with concealed allocation. The study is conducted in a *Maison Sport Santé*, located within a teaching hospital in Grenoble, France. Maisons Sport-Santé are national physical activity facilities in France that aim to promote physical activity and healthy lifestyles for the prevention and management of chronic diseases.

**Fig. 1:**
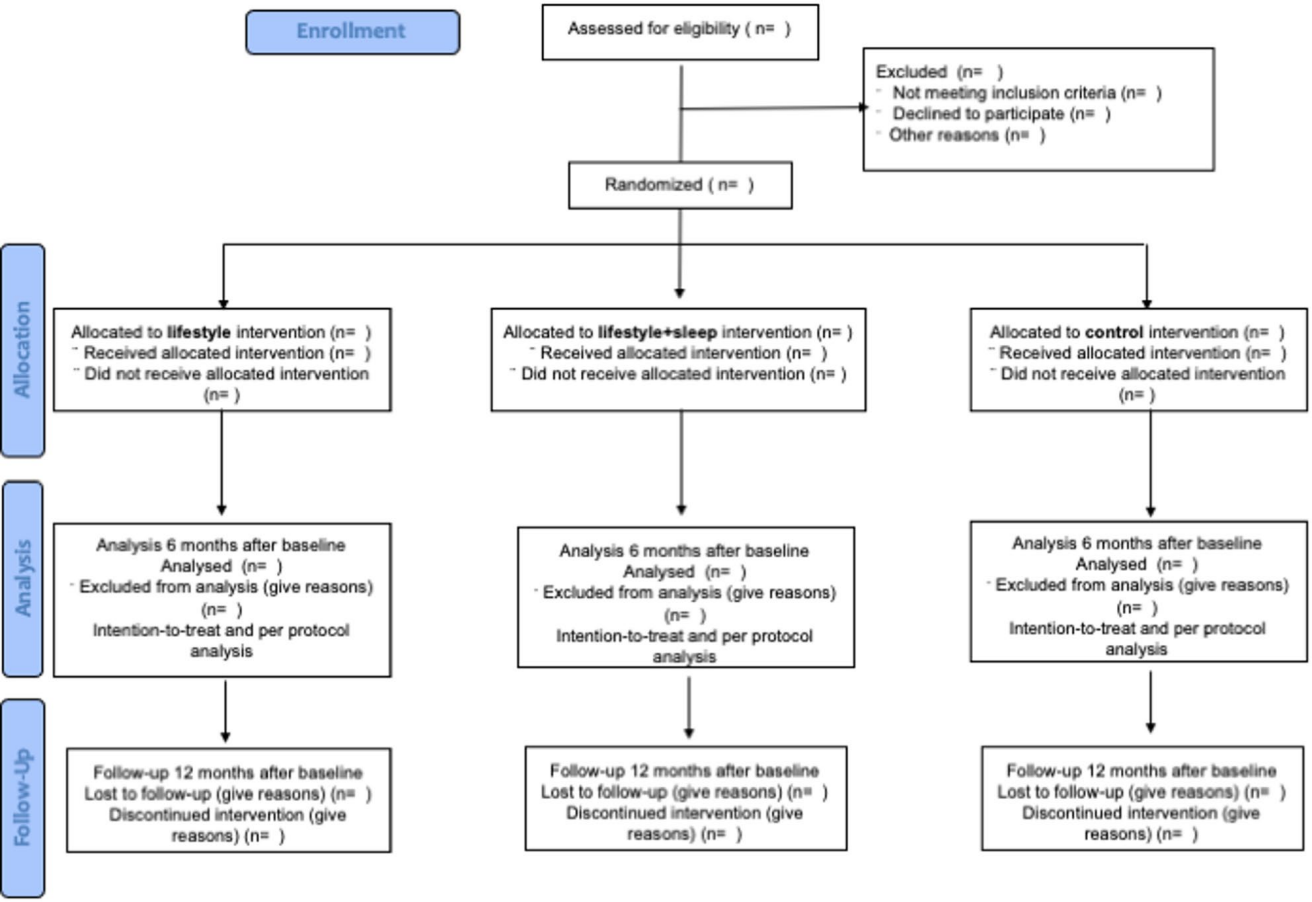
Study design

The intervention is described according to the TIDieR checklist, and the study protocol follows the 2025 SPIRIT guidelines [28] [see Additional file 1 and Table 1]. The conduct and reporting of the trial will comply with CONSORT 2025 guidelines [29]. Assessments will be performed at baseline, post-intervention and after a 6-month follow-up. For an overview of the study design, see Fig. 1.

**Table 1:** Standard Protocol Items: Recommendations for Interventional Trials (SPIRIT) schedule of enrolment, interventions and assessments [28]

TIMEPOINT	STUDY PERIOD					
	Enrollment Pre-study	Visit 1 Baseline t _0_	Visit 2 2 weeks 2 weeks ± 10 days	Visit 3 26 weeks 6 months ± 15 days	Visit 4 52 weeks 12 months ± 15 days	
Enrollment:						
Information	⎫	⎫				
Informed consent		⎫				
Allocation/randomization		⎫				
Interventions:		⎫		⎫	⎫	
Usual Care/Control						
Lifestyle						
Lifestyle + sleep						
Assessments^1^:						
Clinical examination		⎫		⎫	⎫	
Paraclinical examinations		⎫		⎫	⎫	
Notification of adverse events		⎫	⎫	⎫	⎫	
Questionnaires		⎫	⎫	⎫	⎫	
Qualitative interviews				⎫	⎫	

^1^ For the full list of assessments, see outcomes

### Participants

#### Non-inclusion criteria

Participants will be excluded if any of the following apply:

- EQ-5D-5 L score of 11111.
- Presence of any condition that in the opinion of the responsible physician or investigator makes the potential participant unsuitable for the study.
- Diagnosed and treated sleep disturbance (sleep apnea, insomnia, restless legs syndrome) or a sleep consultation conducted within the last year by a sleep specialist (consultation and/or sleep recording via polygraphy or polysomnography).
- Belonging to populations covered in Articles L1121-5, L1121-6 and L1121-8 of the Public Health Code (pregnancy, person deprived of liberty or subject to a legal protection measure, vulnerable person or legally protected adult).
- Current participation in another interventional study.

#### Inclusion criteria

Participants are eligible to participate in the study if they meet the following criteria:

- Aged 18 to 80 years.
- Physically inactive, defined as engaging in less than 150 min of leisure-time physical activity per week.
- Able to provide written informed consent.
- Able to participate in regular physical activity (no medical contraindication to exercise).
- Affiliated to the social security system or benefiting from such a system.

### Recruitment procedure

**Fig. 2:**
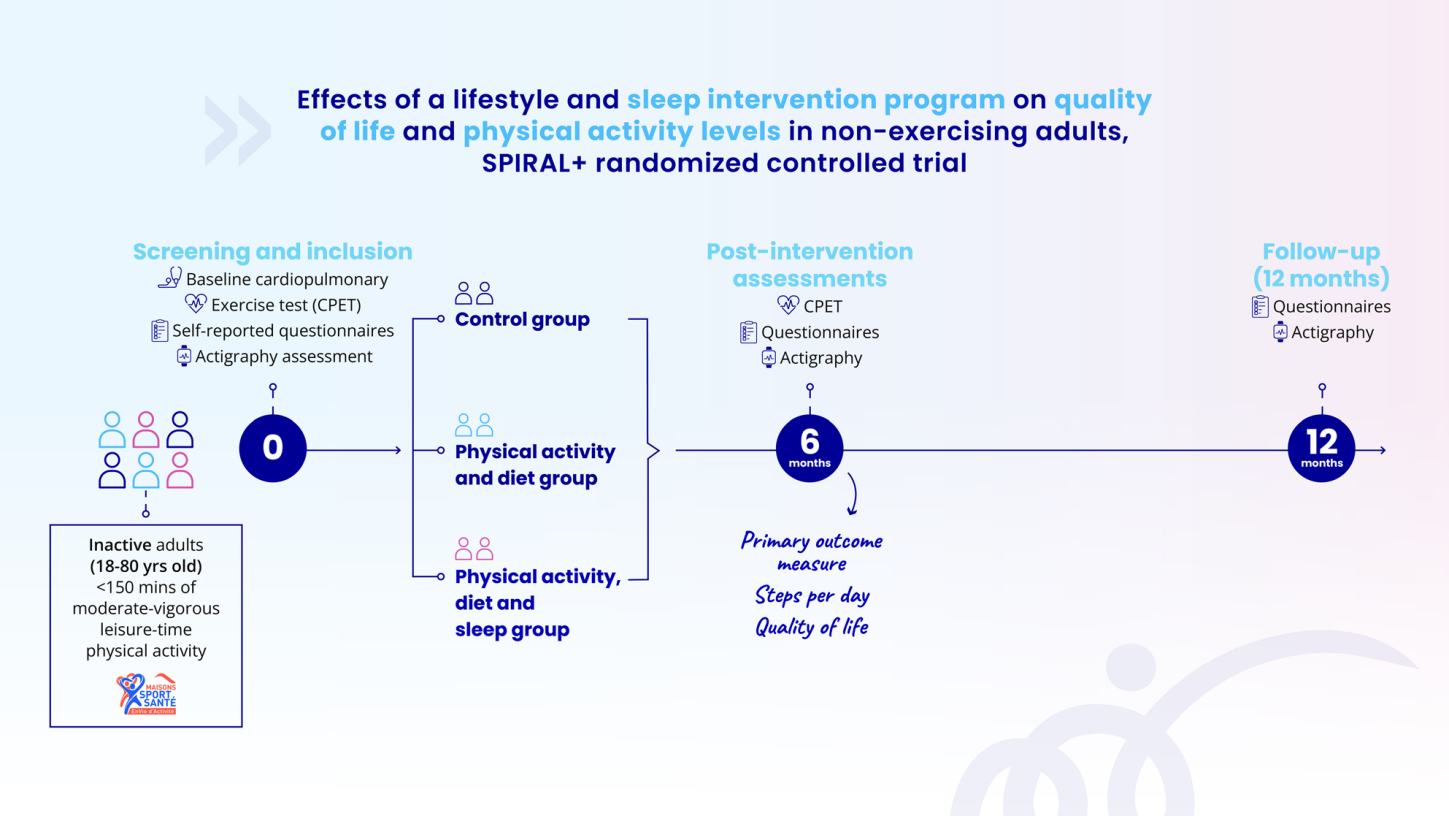
Flowchart of the study design, including enrollment, allocation, and follow-up assessments

The overall trial flow is outline in Fig. 2. Participants will be recruited through passive methods (advertisements in the community and/or on social media) or active methods (through word of mouth or referral through a health care professionals). Community-based recruitment strategies will be employed (local newspapers, radio stations, flyers in medical and paramedical waiting rooms, pharmacies, teaching hospitals, newsletters and contact with potential referral sources). Recruitment started on May 2nd 2024.

### Randomization procedure

People willing to participate and fulfilling the eligibility criteria will be randomized (1:1:1 allocation ratio) after baseline assessment. A priori, an independent statistician will prepare a computer-generated randomization schedule in randomized, permuted blocks of four or six persons.

### Interventions

Participants will be randomized to one of three groups. The first group will receive a 6-month multicomponent lifestyle intervention combining physical activity, diet, a progress journal, and health behavior change workshops. The second group will receive the same lifestyle intervention together with a structured sleep intervention. The third group, serving as the control, will receive standard lifestyle recommendations for physical activity and diet in order to limit deception bias.

#### Lifestyle intervention

Participants randomized to the lifestyle group will participate in a 6-month multicomponent lifestyle intervention. The intervention is informed by Self-Determination Theory and the affectivism approach. The specific components of the physical activity program, the associated behavior change techniques (BCTs) [30], and theoretical constructs are summarized in the additional files [see Additional File 2].

#### Progress journal

At the start of the intervention, participants will be provided with a progress journal to support participant-centered goal setting and behavior tracking throughout the six-month program. This journal includes key information about the lifestyle program. The journal contains an overview of the lifestyle intervention, a weekly physical activity diary for planning and self-monitoring, and sections to identify potential barriers and facilitators. Together with their Adapted Physical Activity (APA) instructor, participants will co-develop weekly challenges aimed at reducing sedentary behavior and/or increasing physical activity.

#### Physical activity component

Participants will be referred to an APA teacher for an individualized 6-month exercise program. The program includes up to 20 supervised sessions (90 min each), combining aerobic and resistance training. On non-supervised days, participants will be encouraged to engage in additional exercise to meet the World Health Organization (WHO) physical activity guidelines (≥ 150 min of moderate-to-vigorous aerobic physical activity and 2 resistance training sessions per week) [1]. Physical activity levels will be objectively monitored throughout the intervention using a wearable device (Withings ScanWatch Light, Withings, France).

#### Diet component

Participants will attend five individual consultations (~ 45 min each) with a registered dietitian. Recommendations will follow French national public health guidelines and be tailored to individual needs and preferences. At the first consultation, the dietitian will conduct a dietary history using a structured checklist to assess usual intake. Food models and metric utensils will be used to estimate portion sizes. Supporting written resources will be provided, including healthy eating guidelines, shopping lists, meal plans, and recipes.

Consultations may address label reading, mindful eating, and food access considerations, depending on participant needs. At each consultation, participants will set personalized goals and discuss barriers and facilitators. The final session will review progress and establish strategies for long-term dietary change.

#### Health-behavior change workshops

Participants will be invited to attend three group-based health behavior change workshops (90 min each) focusing on physical activity and nutrition. The workshops aim to strengthen knowledge about physical activity, sedentary behaviors, and nutrition; explore perceptions of eating patterns; and support participants in setting and planning achievable goals for both physical activity and diet. They also encourage reflection on motivations, strategies to overcome challenges, and the identification of healthy eating behaviors to promote sustainable lifestyle changes.

#### Lifestyle and sleep intervention

Participants randomized to the lifestyle and sleep group will receive the 6-month lifestyle intervention described above, in addition to a structured sleep intervention. The sleep component was developed collaboratively by sleep researchers, certified sleep medicine specialists, and health behavior experts, and is grounded in the Theory of Planned Behavior (TPB) and the Health Action Process Approach (HAPA).

At baseline, a sleep specialist will review participants’ responses to validated questionnaires (Pittsburgh Sleep Quality Index, Epworth Sleepiness Scale, Insomnia Severity Index, Berlin Questionnaire, Horne and Ostberg questionnaire), together with their sleep diary and actigraphy-derived sleep–wake patterns. If further clinical assessment is indicated, participants will be referred for a consultation with a sleep specialist, in collaboration with their general practitioner.

All participants in this group will receive a sleep health program consisting of five individual 30-minute sessions with a trained facilitator. Sessions will cover evidence-based recommendations from the French Institute of Sleep and Vigilance, including: sleep duration and health outcomes, sleep hygiene practices, relationships between sleep and physical activity, and common sleep myths.

Participants will be supported in goal setting and self-monitoring of their sleep behaviors, using both their progress journal and an under-mattress sleep analyzer (Withings Sleep Analyzer, France). Each session will include review of progress, discussion of barriers, and collaborative development of specific, measurable goals for the following period. Examples include regularizing bedtimes, reducing bedroom technology use, or practicing relaxation techniques.

The details of the theoretical framework and the BCTs incorporated in the sleep intervention are summarized in the additional file (see Additional File 2).

#### Control group

Participants randomized to the control group will receive standard lifestyle recommendations (physical activity, diet) in order to limit deception bias.

### Outcomes

#### Primary outcome

The study has two primary outcomes, assessed 6 months after randomization:

1. Steps per day, measured objectively using an Actigraph GT3X accelerometer (Actigraph Corp, USA). Steps per day were chosen because increases in daily step count are strongly associated with reduced all-cause mortality [31]. Furthermore, step count is passively tracked by widely used consumer technologies (e.g., smartphones), giving it practical relevance. Recent dose–response evidence also provides insights to inform future physical activity guidelines [32].
2. Health-related quality of life, measured with the EQ-5D-5L questionnaire. This instrument is a widely used, cognitively simple, self-administered measure that assesses five dimensions of health (mobility, self-care, usual activities, pain/discomfort, anxiety/depression), each rated on five severity levels, along with a visual analog scale (EQ-VAS) for overall health [33]. Quality of life was selected as a co-primary outcome because self-rated health and health-related quality of life are robust predictors of mortality and chronic disease risk, and are sensitive to changes in lifestyle behaviors.

### Secondary outcomes

A range of secondary outcomes will also be assessed at baseline and different study timepoints (see Table 2).

**Table 2:** SPIRAL+ Study Data Collection and Outcome Measures

Variable	Instrument	Baseline (T0)	Follow-up visit (2 weeks)	End of intervention (26 weeks)	12-month Follow-up (52 weeks)
Anthropometric & clinical data					
Waist, weight, height, BMI, blood pressure	Height to nearest 0.1 cm. Body weight to nearest 0.1 kg. BMI = weight divided by height squared kg/m^2^ Resting BP with sphygmomanometer	⎫		⎫	⎫
Body composition	Bio-electrical impedance	⎫		⎫	⎫
Device-measured physical activity	Actigraph GT3X	⎫		⎫	⎫
Device-measured sedentary time	Actigraph GT3X				
Device-measured sleep	Actigraph GT3X	⎫		⎫	⎫
Sleep	Sunrise sleep device	⎫		⎫	
Cardiorespiratory fitness	VO_2peak_	⎫		⎫	
Handgrip strength	Dynamometer	⎫		⎫	⎫
Self-report (questionnaires)					
Health-related quality of life	EQ-5D-5 L	⎫		⎫	⎫
Self-reported physical activity	Global Physical Activity Questionnaire (GPAQ)	⎫		⎫	⎫
Diet quality	Cardiovascular Diet Questionnaire (CDQ-2)	⎫		⎫	⎫
Sleep quality	Pittsburgh Sleep Quality Index (PSQI)	⎫		⎫	⎫
Quality of life	Quality of life SF-36	⎫		⎫	⎫
Wellbeing	Warwick–Edinburgh Mental Wellbeing Scale (34)	⎫		⎫	⎫
Sleepiness	Epworth Sleepiness Scale	⎫		⎫	⎫
Insomnia symptoms	Insomnia Severity Index	⎫		⎫	⎫
Chronotype	Horne Ostberg Morning Eveningness Questionnaire	⎫		⎫	⎫
Motivation	Motivation Scale towards Health-Oriented Physical Activity (EMAPS)	⎫		⎫	⎫
Preference for and tolerance of exercise intensity	Preference for and Tolerance of Exercise Intensity Questionnaire	⎫		⎫	⎫
Attitude self-efficacy subjective norms and intention	Attitude self-efficacy subjective norms and intention	⎫		⎫	⎫
Vitality	Subjective vitality	⎫		⎫	⎫
Physical Activity Regulation	The Physical Activity Regulation Scale			⎫	
Competence, autonomy, relatedness	The Basic Need Satisfaction Scale			⎫	
Behaviors of the APA teacher	The Interpersonal Behaviors Questionnaire			⎫	
Implementability of the interventions	Acceptability, fidelity, feasibility			⎫	
Program adherence	The number of APA/diet/health-behavior workshops and sleep sessions attended, divided by total number of sessions			⎫	

### Clinical and paraclinical parameters

#### Cardiopulmonary exercise test

All participants will perform a graded maximal exercise test to determine peak O_2_ uptake (VO_2_) and parameters to guide aerobic exercise prescription. Participants will pedal on a cycle ergometer and wear a mouthpiece and nose-clip that permits breath-by-breath measurement of minute ventilation, O2 uptake and CO_2_ production from expired gas. Power output is increased every minute until the patient indicates either that he/she would like to stop, a physiological maximum is achieved (indicated by heart rate, BP, respiratory exchange ratio or VO_2_ plateau) or abnormalities appear that necessitate discontinuation. Work rate, heart rate, electrocardiogram and blood pressure data will be collected.

#### Body composition

Body composition will be determined using bioelectrical impedance. Bioelectrical impedance analysis is a method for estimating body composition, in particular body fat and muscle mass, where a weak electric current flows through the body and the voltage is measured in order to calculate impedance (resistance and reactance) of the body. Bioelectrical impedance will be measured using a bioelectrical impedance spectrum analyser (SFB7, Impedimed, Impedimed Ltd.).

#### Handgrip test

The purpose of the handgrip strength test is to measure the maximum isometric strength of the hand and forearm muscles. The participant holds the dynamometer in the hand to be tested, with the arm at right angles and the elbow by the side of the body. The handle of the dynamometer is adjusted if required - the base should rest on the first metacarpal (heel of palm), while the handle should rest on middle of the four fingers. When ready the participant squeezes the dynamometer with maximum isometric effort, which is maintained for about 5 s. No other body movement is allowed. The participant is be strongly encouraged to give a maximum effort. Three repetitions are performed, each separated by a 30-second rest period, and the best value is retained for analysis.

### Physical activity, sedentary behavior and sleep

Baseline physical activity and sedentary time will be evaluated with an Actigraph monitor (Actigraph GT3X, Actigraph Corp). Patients will be equipped with a research-grade activity monitor and will be asked to wear it for 7 days. The Actigraph is a small (5.1 × 4.1 × 1.5 cm), light (0.042 kg) instrument that records integrated acceleration information as an activity count, which provides an objective estimate of the intensity of body movements. The device is programmed to record in 1-minute epochs. Participants will be instructed to wear the monitor on their non-dominant wrist during all waking hours. However, because the monitors are not waterproof, participants are asked to take them off while bathing or swimming. They will also complete a sleep diary in order to indicate time in bed and time out of bed.

#### Home sleep test

Sleep will be evaluated using a disposable medical device that is non-invasive and has CE marking (class IIa, under the European Directive 93/42/CEE). This device is developed by SUNRISE SA, and can be used in the home. The device is based on an automated mandibular jaw movement analysis (Sunrise, Namur, Belgium). It comprises a single point of contact sensor weighing 3 g that is attached to the *mentolabial sulcus* of the individual. It includes an embedded inertial measurement unit that records the rotational movement of the mandible using a gyroscope and the position of the mandible using an accelerometer. The sensor communicates with a smartphone application for external control and enables mandibular jaw movement data transfer overnight via Bluetooth. Recorded raw data is then transferred to a cloud-based infrastructure at the end of the night using a secured data protocol, where it is pre-processed and automatically analyzed by a machine learning-based algorithm.

### Questionnaires

Participants will complete validated questionnaires covering physical activity, sleep, diet, wellbeing, and motivational/psychological constructs, including:

- *Global Physical Activity Questionnaire (GPAQ)*: The GPAQ is a questionnaire developed by the World Health Organization (WHO) to assess physical activity levels in diverse populations. It measures physical activity across three domains: Work-related activity (occupational or domestic tasks, Travel-related activity (walking or cycling for transportation), Leisure-time activity (exercise, sports, or recreational activities). The GPAQ also captures sedentary behavior and provides data on the frequency, intensity, and duration of activities.
- *Pittsburgh Sleep Quality Index (PSQI)*: The PSQI is a self-reported questionnaire that assesses sleep quality and quantity in terms of seven components, namely, subjective sleep quality, sleep latency, sleep duration, habitual sleep efficiency, sleep disturbances, use of sleeping medications, and daytime dysfunction (Buysse, Reynolds, Monk, Berman, & Kupfer, 1989).
- *Warwick–Edinburgh Mental Wellbeing Scale (WEMWS).* This WEMWS is a 14-item scale of positively worded statements covering feeling and functioning aspects of mental wellbeing [34]. The 14-statements have five response categories from ‘none of the time’ to ‘all of the time’.
- *Epworth Sleepiness Scale (ESS)*: The ESS measures daytime sleepiness and consists of eight items (situations) where individuals assess how likely they would fall asleep [35].
- *Berlin questionnaire*: The Berlin questionnaire is a self-administered questionnaire that was developed to identify subjects with obstructive sleep apnea (OSA) in primary care settings [36].
- *Horne Ostberg Morning Eveningness Questionnaire (MEQ)*: The MEQ consists of 19 items and was developed to assess individual differences in morningness and eveningness – the degree to which respondents are active and alert at certain times of day. Scale items query preferences in sleep and waking times, and subjective “peak” times at which respondents feel their best.
- *Insomnia Severity Index (ISI)*: The ISI is a 7-item self-report questionnaire assessing the nature, severity, and impact of insomnia [37]. The dimensions evaluated are: severity of sleep onset, sleep maintenance, and early morning awakening problems, sleep dissatisfaction, interference of sleep difficulties with daytime functioning, noticeability of sleep problems by others, and distress caused by the sleep difficulties.
- *Quality of life (SF-36)*: The Medical Outcomes Study 36-item Short-Form Health Survey (SF-36) is a widely used, generic, patient-report measure created to assess health-related quality of life in the general population. The SF-36 measures eight scales: physical functioning, role physical, bodily pain, general health, vitality, social functioning, emotional role, and mental health. Component analyses showed that there are two distinct concepts measured by the SF-36: a physical dimension, represented by the Physical Component Summary (PCS), and a mental dimension, represented by the Mental Component Summary (MCS).
- *Cardiovascular Diet Questionnaire (CDQ-2)*: This CDQ-2 is validated against the reference method (7-day dietary history) and with biomarkers including plasma contents of saturated fatty acids (SFA), unsaturated fatty acids (UFA), UFA/SFA ratios and plasma folate concentration was used as a biomarker of the intake of fruits and vegetables [38]. It consists of 17 closed-ended questions designed to capture the main sources of the relevant nutrients.
- *Motivation Scale towards Health-Oriented Physical Activity (EMAPS)*: The EMAPS is an 18-item scale designed to measure self-determined motivation toward physical activity [39]. It measures 6 dimensions of motivation: intrinsic motivation, identified regulation, integrated regulation, introjected regulation, external regulation, and amotivation.
- *Preference for and Tolerance of Exercise Intensity* Questionnaire (PRETIE-Q): The PRETIE-Q is a 16-item scale designed to measure the preference for and tolerance of exercise intensity [40].
- *The Physical Activity Regulation Scale (PARS)*: The PARS is a 14-item scale designed to measure behavioural regulation tactics used to manage actions after the formation of a physical activity intention. It comprises four dimensions: (a) proactive regulation, (b) reactive regulation, (c) social monitoring, and (d) self-monitoring [41].
- *The Basic Need Satisfaction Scale* : The Basic Need Satisfaction Scale is 15-item scale designed to measure the need for autonomy, the need for competence, and the need for relatedness [42].
- *The Interpersonal Behaviors Questionnaire (IBQ)*: The IBQ is a 24-item scale designed to measure the behaviors of the Adapted Physical Activity (APA) teacher. It comprises 6 dimensions: autonomy support, autonomy hindering, competence support, competence hindering, relatedness support, and relatedness hindering [43].

#### Secondary qualitative objectives

We also aim to explore the profiles of participants who refuse to participate, who are non-adherent to the intervention programs and who also who adhere fully to the programs in order to decipher the role played by physical, environmental, sociological and psychological factors from a transdisciplinary perspective. The objective will be to obtain a better understanding of adherence to physical activity interventions and recommendations and to identify potential factors which were not identified in the quantitative analysis. For this purpose, we aim to:

1. Examine the links made by participants who are non-adherent regarding the different factors associated with non-adherence to physical activity. Understand how these individuals explain these situations on the basis of their personal situation, their representation of health and/or their understanding of the impact of physical activity on health.
2. Understand how these individuals explain these situations, in regard to the program adherence, on the basis of their personal situation, their representation of health and/or their understanding of the impact of physical activity on health.
3. For adherent patients, to explore the factors which are associated with the success to the program.
4. To understand the interactions between patients and healthcare professional in the conduct of the program and the success or failure.

#### Secondary qualitative outcome measures

Criteria will come from the analyses of semi-structured qualitative interviews conducted separately with participants and healthcare professional. These interviews will explore participants’ experience with physical activity and exercise, motivation to perform physical activity, beliefs that exercise will improve health, factors that limit their ability to engage in exercise, and the importance of self-regulation in exercise adherence.

#### Implementability of the intervention

The criteria of acceptability, fidelity, and feasibility are evaluated according to the analytical framework of Klaic et al. [44]. Acceptability, that is, the extent to which the individuals who deliver or receive the intervention consider it appropriate, will be assessed using the following indicators: the number of study dropouts, participant satisfaction; and adverse effects reported by professionals and participants.

Fidelity, understood as the strategies used to control and improve the reliability and validity of the intervention, will be evaluated using the following indicators: the fidelity of the intervention to participants’ expectations; responsiveness to participants’ needs; the quality of interpersonal behaviors of professionals as perceived by participants, assessed with the Interpersonal Behavior Questionnaire (IBQ).

Feasibility will be assessed based participant adherence to the intervention, i.e., the number of individuals attending the scheduled sessions, will be recorded, along with the reasons for non-adherence as reported by participants to the coordinator.

### Sample size calculations

Sample size and power calculations were performed using R statistical software under the following assumptions: balanced randomization (1:1:1 across the three arms); changes in steps per day follow a Gaussian distribution, centered at 0 in the control arm, 1800 in the lifestyle intervention arm, and 2200 in the lifestyle and sleep intervention arm, with a standard deviation of 2121 [45, 46]; changes in EQ-5D-5 L scores follow a Gaussian distribution, centered at 0 in the control arm, 0.07 in the lifestyle intervention arm, and 0.14 in the lifestyle and sleep intervention arm [47], with a standard deviation of 0.21. This 0.07 increase is based on the anticipated observation of a minimal important difference to infer changes in health-related quality of life [48].

Based on these assumptions, a sample of 181 participants provides > 90% power to detect the following three primary objectives:

(i) superiority of the lifestyle arm versus control for steps per day;
(ii) superiority of the lifestyle + sleep arm versus control for steps per day;
(iii) superiority of the lifestyle + sleep arm versus control for EQ-5D-5 L.

This design also provides reasonable power for two additional comparisons:

(iv) lifestyle versus control for EQ-5D-5 L;
(v) lifestyle + sleep versus lifestyle for EQ-5D-5 L.

Accounting for an anticipated 10% dropout rate, the study will aim to recruit 201 participants.

### Data analysis

The full statistical analysis plan will be validated before the database is locked, which will be performed according to the RCDMS.MOP.009 operating procedure of the CHU Grenoble Alpes, after the usual data management procedure and according to the specifications defined a priori. The general approach of the data analysis is described below:

Outcomes will be compared between groups following the intention-to-treat (ITT) principle, i.e. all patients will be analyzed in the group they were initially allocated to. However, this implies obtaining complete data for all randomized patients, which in practice is rarely possible. For the main analysis, we propose an analysis on the modified ITT population (mITT), which will include patients for whom the following protocol deviations will be observed:

- Patients assigned to an intervention group who finally did not receive the (or received incomplete) intervention, whatever the reason, but who completed follow-up, will be analyzed in the corresponding intervention group;
- Patients allocated to the control group who would benefit from another intervention will be analyzed in the control group;

In addition, we propose the following two sensitivity analyses. First, an analysis on the ITT population, i.e. including patients who did not complete follow-up. This implies missing data imputation. Multiple imputation methods will be used, provided the assumption that the data are missing at random (MAR) is credible [49, 50]. If the ‘missing not at random’ (MNAR) hypothesis cannot reasonably be excluded, different sensitivity analyses (best-worst and worst-best) will be implemented to estimate the extent of the uncertainty associated with these missing data. Second, an analysis on the per protocol population, i.e. patients who actually received the treatment as initially allocated, and who completed follow-up.

The characteristics of the study population at inclusion will be presented by the mean and standard deviation for continuous variables (or by the median and interquartile range, depending on their distribution); and by their frequency and percentage for categorical variables.

#### Analysis of the outcomes

The primary objectives consist in 5 different questions, which are organized in a single hierarchy (see Figure S1) so as to control the overall type I error of the primary objectives at a 5% level. Therefore, to avoid the inflation of alpha risk associated with multiple tests, the next level of the hierarchy will only be tested if the null hypothesis of the previous level is rejected. Primary and secondary outcomes (continuous variables) will be compared between groups using a t-test. Mixed effects linear models will also be used when necessary (repeated measures). The significance level of all tests corresponding to secondary objectives will be set at 0.05. Analyses will be conducted using R software or SAS.

### Patient and public involvement

Healthcare professionals and community stakeholders were consulted during the development of this lifestyle intervention to ensure its feasibility and relevance in real-world settings. Their input primarily helped identify key barriers to behavior change, such as time constraints, accessibility issues, and common misconceptions about sleep, physical activity, and diet.

While direct involvement from the target population was limited, discussions with professionals familiar with these individuals’ needs guided adjustments to the intervention’s structure and delivery. For example, recommendations were made regarding the practical integration of behavior change strategies into daily life and the potential challenges participants might face in maintaining long-term engagement.

Public involvement in later stages of the study will remain minimal, however findings will be shared with relevant stakeholders, including healthcare professionals and local organizations, to support the potential adaptation of the intervention for broader implementation.

### Dissemination of project findings

The findings of this study will be disseminated through multiple channels to ensure they reach both academic and practical audiences. Results will be published in peer-reviewed journals and presented at scientific conferences focused on health behavior change, physical activity, nutrition, and sleep. Additionally, targeted presentations will be conducted for healthcare professionals and community stakeholders to facilitate the integration of evidence-based strategies into practice. Where possible, summaries of key findings will be shared in accessible formats, such as infographics or brief reports, to engage a wider audience. By leveraging these dissemination strategies, we aim to maximize the impact of our findings and contribute to the development of more effective lifestyle interventions in both clinical and community settings.

## Discussion

To our knowledge, this is the first fully powered trial to investigate the effectiveness of a lifestyle intervention integrating sleep, physical activity, and diet on quality of life and physical activity levels in inactive adults. This study builds on previous research that has demonstrated the individual benefits of physical activity and diet interventions, but has yet to examine the combined impact of these factors alongside sleep improvement in this population.

The SPIRAL+ study aims to fill significant gaps in the existing evidence on lifestyle interventions. While there is substantial research on the individual effects of physical activity, diet, and sleep, there is limited evidence on how a multi-component intervention targeting all three behaviors may work together to improve long-term health outcomes in inactive adults. By including a sleep component, this study addresses the critical need for interventions that focus on improving sleep quality, which is often overlooked despite its significant role in overall health and well-being.

A notable strength of this trial is its longitudinal design, with assessments at both 6 and 12 months. The 6-month timepoint allows us to evaluate the immediate effectiveness of the intervention, while the 12-month follow-up provides insight into the sustainability of behavioral changes and their long-term impact on health outcomes. This extended follow-up is particularly important given that maintaining improvements in physical activity, diet, and sleep remains a major challenge in lifestyle interventions.

Methodologically, the study combines objective and subjective assessments of both physical activity and sleep, providing a comprehensive evaluation of the intervention’s impact. Similarly, the addition of validated questionnaires offers insights into diet, mental wellbeing, and motivational processes, capturing psychological and behavioral dimensions that may explain adherence and maintenance. The qualitative component further strengthens the study by exploring barriers and facilitators to engagement, which may inform strategies to enhance scalability and real-world implementation.

In conclusion, if the lifestyle and sleep intervention proves to be effective in improving quality of life and increasing physical activity levels, it could have significant implications for public health strategies. The incorporation of sleep into lifestyle interventions may become a crucial component for promoting sustained health improvements, with the potential for broad-scale implementation.

## Supplementary Information

Supplementary Material 1.

Supplementary Material 2.

## Data Availability

No datasets were generated or analysed during the current study.

## Abbreviations

APA: Adapted Physical Activity
BCTs: Behavior Change Techniques
BMI: Body Mass Index
BP: Blood Pressure
CDQ-2: Cardiovascular Diet Questionnaire–version 2
CE: Conformité Européenne (European Conformity)
CHU: Centre Hospitalier Universitaire
CONSORT: Consolidated Standards of Reporting Trials
EQ-5D-5L: EuroQol 5–Dimension 5–Level questionnaire
EQ-VAS: EuroQol Visual Analog Scale
EMAPS: Échelle de Motivation envers l’Activité Physique en Santé (Motivation Scale towards Health–Oriented Physical Activity)
ESS: Epworth Sleepiness Scale
GPAQ: Global Physical Activity Questionnaire
HAPA: Health Action Process Approach
HBC: Health Behavior Change
IBQ: Interpersonal Behaviors Questionnaire
ISI: Insomnia Severity Index
ITT: Intention–to–Treat
MAR: Missing At Random
MCS: Mental Component Summary (SF–36)
MEQ: Morningness–Eveningness Questionnaire (Horne–Östberg)
mITT: Modified Intention–to–Treat
MNAR: Missing Not At Random
OSA: Obstructive Sleep Apnea
PARS: Physical Activity Regulation Scale
PCS: Physical Component Summary (SF–36)
PRETIE-Q: Preference for and Tolerance of Exercise Intensity Questionnaire
PSQI: Pittsburgh Sleep Quality Index
RCT: Randomized Controlled Trial
SAS: Statistical Analysis System (software)
SFB7: Segmental Bioelectrical Impedance Analyzer (model SFB7, Impedimed)
SDT: Self–Determination Theory
SF-36: 36–Item Short Form Health Survey
SPIRAL+: SleeP exercIse nutRition heALth+ study
SPIRIT: Standard Protocol Items: Recommendations for Interventional Trials
TIDieR: Template for Intervention Description and Replication
TPB: Theory of Planned Behavior
VO₂: Oxygen Uptake
WEMWBS: Warwick–Edinburgh Mental Wellbeing Scale
WHO: World Health Organization
