# Supplementary material for: Study protocol for a theory-informed randomized controlled trial of a lifestyle and sleep intervention to improve quality of life and physical activity in inactive adults: the SleeP exercIse nutRition heALth+ SPIRAL+ study
Source: BMC Public Health. 2026 Mar 13;26:1297. doi: 10.1186/s12889-026-26959-4 (PMC13097937; doi:10.1186/s12889-026-26959-4)
Supplement: Supplementary file 1 — Supplementary Material 1. [file 12889_2026_26959_MOESM1_ESM.docx]

**Additional File 1. Theoretical framework and behavior change techniques in the lifestyle intervention**

Self-determination theory is a theory of human motivation that has demonstrated efficacy in predicting motivated behavior in multiple contexts and populations, and for a variety of behaviors (20, 21), including health behaviors such as physical activity, healthy eating, and smoking cessation (22).

The self-determination theory (SDT) is a well-established framework that examines the factors supporting sustained motivation and well-being **(20).**  At its core, this model describes motivation as existing along a continuum, ranging from the absence of motivation to fully autonomous motivation, where behavior is driven by personal choice and volition. Research indicates that autonomous motivation leads to positive emotional, cognitive, and behavioral outcomes and is strongly linked to long-term physical activity engagement. The most autonomous forms of motivation are intrinsic, arising when individuals engage in an activity for its inherent enjoyment and personal fulfillment. In particular, participating in physical activity for the pleasure and satisfaction it brings is a key predictor of long-term adherence **(23)**. Other forms of autonomous motivation may arise when behavior engagement aligns with one’s identity (integrated regulation) or personal values (identified regulation). These forms of autonomous motivation strengthen when three fundamental psychological needs are met: autonomy (the need to feel in control of one’s actions), competence (the need to feel capable and effective in interacting with the environment), and relatedness (the need to feel connected to others) (21). Recently, a classification of motivation and behavior change techniques has been proposed to better explain how these theoretical principles can be translated into practice (52).

In addition to grounding our intervention in SDT, we also drew on recent research highlighting the role of affective responses in physical activity engagement and maintenance (53). This line of research suggests that affective experiences during physical activity may influence future behavior by shaping both remembered pleasure and anticipated pleasure in similar contexts (54). More specifically, the affective experience depends on the intensity of exercise: (1) low intensities (below ventilatory threshold, VT) result in a positive affective response; (2) the affective response to the intensities at VT is heterogeneous – pleasant for some and unpleasant for others; (3) high intensities (above VT) produce negative affective response (Ekkekakis, Hall, & Petruzzello, 2008). However, not all aspects of an experience are equally influential in shaping the global affective evaluation of that experience. The peak- and end-rule (Fredrickson, 2000; Schreiber & Kahneman, 2000), posits that the most intense affective moment (the “peak”) and the final affective moment (the “end”) disproportionately influence how the overall experience is remembered and whether it is repeated (24).

This highlights that a multi-behavior lifestyle intervention fostering positive emotional experiences and supporting the three fundamental psychological needs could enhance participants’ autonomous motivation, ultimately promoting sustained health-enhancing behaviors (i.e. physical activity and diet).

| BCT | Related feature of the lifestyle intervention | Theoretical framework |
| --- | --- | --- |
| Goal setting, behavior (1.1) | ***Initial meeting for PA or diet***  Set person-centered goals regarding their health behaviors and write them in the progression journal.  ***Health-behavior change workshops***  Choose a goal regarding physical activity and diet. | **SDT (competence)** |
| Goal setting, outcome (1.3) | ***Initial meeting for PA or diet***  Set a goal defined in terms of a positive outcome of targeted health behavior  Agree on weekly goals and write them in the progression journal | **SDT (competence)** |
| Action planning (1.4) | ***During the PA sessions***  Plan the weekly PA session (with details about context, frequency, duration, intensity), and write it down in the progression journal.  Prompt planning of PA sessions that the participant will do on their own, and write them in the progression journal | **SDT (competence)** |
| Review behavior goals (1.5) | ***During PA sessions or with the dietician***  Review behavior goal(s) jointly with the person and consider modifying goal(s) or behavior change strategy in light of achievement. This may lead to re-setting the same goal, a small change in that goal or setting a new goal instead of (or in addition to) the first, or no change.  Invite the participant to write it in the progresion journal. | **SDT (competence)** |
| Discrepancy between current behavior and goal standard (1.6) | ***Health behavior change workshops***  Draw attention to discrepancies between a person’s current behavior (in terms of the form, frequency, duration, or intensity of that behavior) and the person’s previously set outcome goals, behavioral goals or action plans (goes beyond self-monitoring of behavior).  Help the person identify barriers and facilitators to resolve problems. | **SDT (competence)** |
| BCT 1.7 Review of outcome goal(s) | ***During PA sessions or with the dietician***  Review outcome goal(s) jointly with the person and consider modifying goal(s) in light of achievement. This may lead to re-setting the same goal, a small change in that goal or setting a new goal instead of, or in addition to the first | **SDT (competence)** |
| BCT 2.2 Feedback on behavior | **PA**  Inform about the level of achievement of the previously set objectives using the progress journal. | **SDT (competence)** |
| TCC 2.3 Self-monitoring of behavior | **PA session**  Self-monitor physical activity and goals on the digital platform. | **SDT (autonomy)** |
| BCT 2.6 Biofeedback with physiological parameters (i.e. HR) | **PA session**  Provide information regarding improvement in health related parameters (i.e. HR during aerobic exercise). | **SDT (competence)** |
| BCT 4.1 Instruction on how to perform a behavior | **PA session**  Videos that provide instructions on exercises, available on the digital platform. | **SDT (competence)** |
| BCT 6.1 Modeling of the behavior | **PA session**  Videos that provide demonstrations of the exerices. | **SDT (competence)** |
| BCT 8.7 Graded tasks | **PA session**  Individualize the program so that there is an increment in the level of difficulty based on the participants capacities.  Proposer un programme d’AP avec une incrémentation de la difficulté en fonction des capacités du participant. | **SDT (competence)** |
| MBCT 6. Provide choice | **PA session**  Propose the choice of activity  Propose the choice of exercises during the session  Propose exercise variations to adjust the difficulty (e.g., push-up, push-up on knees, inclined push-up)  Encourage self-regulation of the number of sets, duration, and exercise intensity | **SDT (autonomy)** |
|  |  |  |
| MBCT 5. Provide a meaningful rationale | **PA session**  **Provide rational explanations for the usefulness of the exercise** (e.g., explain which muscle groups are targeted; what daily improvements can be expected). | **SDT (autonomy)** |
|  |  |  |
| MBCT 3. Use non-  controlling, informational  language | **PA session**  Use the conditional with phrases like "you could" rather than expressions that imply pressure or guilt, such as "you must" or "you have to." | **SDT (autonomy)** |
| MBCT 17. Assist in  setting optimal challenges | **PA session**  Communicate clear session objectives at the beginning of the session  Co-define individualized weekly challenges and record them in the progress journal  Offer activities that can be adapted to each person's abilities, with a progression in complexity/difficulty throughout the program | **SDT (competence)** |
| MBCT 18. Offer constructive, clear, and relevant feedback | **PA session**  Offer feedback on progress and objectives  Provide individualized solutions and advice. | **SDT (competence)** |
| MBCT 20. Promote self-monitoring | **PA session**  Encourage participants to use the logbook to record both supervised and independent activities, as well as progress in objectives and challenges  Provide self-monitoring tools (e.g., wearables) | **SDT (competence)** |
| MBCT 10. Show unconditional regard | **PA session**  Express unconditional positive support. | **SDT (relatedness)** |
| MBCT 14. Prompt identification and seek available social support | **PA session**  Help identify people in the social circle (e.g., family, friends) to practice or support engagement in physical activities. | **SDT (relatedness)** |
| Allow self-regulation of exercise intensity. | **PA session**  Encourage experimentation and the use of the enjoyment scale to self-regulate exercise intensity. | **Affective response to exercise** |
| PA session | **PA session** | **Affective response to exercise** |
| Propose a happy ending. | **PA session**  At the end of the session, propose a cool-down and a playful activity. | **Affective response to exercise** |

##### **Additional File 2. Theoretical framework and BCTs implemented in the sleep intervention**

According to theory of planned behavior (TPB), behavioral intention—an individual’s expression of their decisions to perform or not perform a specific behavior—is the most proximal determinant of an individual’s behavior. Behavioral intention, in turn, is determined by three constructs: attitudes toward the behavior (ie, whether an individual prefers or values the specific behavior), subjective norms (ie, the opinions of the individual’s significant others on the specific behavior), and perceived behavioral control (ie, how confident the individual feels in performing the specific behavior) (26). The TPB has been used for understanding a variety of behaviors including sleep hygiene behaviors (25, 26). Based on TPB, health care providers may try to improve the attitudes, subjective norms, and perceived behavioral control of individuals on their sleep hygiene behaviors. Subsequently, the individual may have elevated intention to practice good sleep hygiene.

The TPB is, however, limited in that it does not consider the process(es) by which intentions are translated into action. Given the widely cited gap between intention and action, models such as the HAPA build on the motivational constructs specified by the TPB to consider self-regulatory processes like action and coping planning that might help us to explain why some intentions are translated into action, while others are not.

The HAPA suggests that changing behavior (e.g. improving sleep hygiene in an effort to get more sleep) involves two consecutive phases: (1) a motivational phase and (2) a self-regulatory phase (27). The self-regulatory phase of the HAPA suggests that action planning and coping planning are likely to be important in determining whether intentions are translated into action. That is, after an individual forms the intention to perform health behaviors (e.g. “I *will try to go to sleep at the same time each evening*”), action planning helps him or her to plan when, where, and how to perform such behaviors (e.g. “*I will go to bed after I watch the 10pm new*s”) and coping planning helps him or her to design strategies to overcome anticipated barriers to performing such behaviors (e.g. “*If I am tempted to look at my phone before bed, then I will tell myself that it will still be there in the morning.*”).

Again, a behavior change technique taxonomy has been developed to better translate principles issued from these theories to practice (55).

| BCT | Brief description | Targeted outcome(s) |
| --- | --- | --- |
| Information about health consequences (5.1) | Explain that adults need between 7 and 9 hours of sleep every day to function best.  *According to the INSV.*  Less sleep (<7 hrs) can affect the CDV, endocrine, immune and nervous systems, including anxiety, depression, obesity and impaired glucose tolerance  ***Session 1*** | Attitude towards and intentions to perform sleep hygiene behaviors |
| Pros & cons (9.2) | Outline the potential risks of insufficient sleep (BP, stroke, diabetes, CDV).  The participants are asked to list the potential risks of poor sleep and the potential benefits of goof sleep  ***Session 1*** | Attitudes toward and intentions to perform sleep hygiene behaviors |
| Reconstructiong the physical environement (12.1) | Recommendations for making the bedroom more comfortable for sleeping (e.g. keep the temperature in your bedroom comfortable)  ***Session 1*** | Perceived behavioral control |
| Action planning (1.4) | Ask participants to plan actions specifying what (sleep hygiene behaviors), when (day), and where (place) they would sleep  ***Session 1, 2, 3, 4*** | Action planning |
| Problem solving (1.2) | Ask participants to identify barriers that they may encounter when trying to sleep and generate strategies to overcome them. For example : « If I can’t sleep because I am worried about work, then I will try a relaxation technique »  ***Session 1, 2, 3, 4*** | Coping planning |
| Self-monitoring of behavior (2.3) | Participations will be provided with a Sleep Analyzer that will allow them to track their sleep (timing, regularity, social jetlag)  ***Throughout the intervention*** | Self-monitoring |
